# Natural products for the treatment of depression: Insights into signal pathways influencing the hypothalamic–pituitary–adrenal axis

**DOI:** 10.1097/MD.0000000000035862

**Published:** 2023-11-03

**Authors:** Jiawen Liu, Tianwei Meng, Chaojie Wang, Weiping Cheng, Qi Zhang, Guangyu Cheng

**Affiliations:** a Graduate school, Heilongjiang University of Chinese Medicine, Harbin, Heilongjiang, China; b The Second Ward of Acupuncture and Moxibustion Department, First Affiliated Hospital, Heilongjiang University of Chinese Medicine, Harbin, China; c The Forth Ward of Cardiovascular Department, First Affiliated Hospital, Heilongjiang University of Chinese Medicine, Harbin, China; d The Sixth Ward of Acupuncture and Moxibustion Department, First Affiliated Hospital, Heilongjiang University of Chinese Medicine, Harbin, China.

**Keywords:** action mechanism, depression, hypothalamic–pituitary–adrenal axis, natural medicines, traditional Chinese medicine

## Abstract

Depression, a prevalent psychiatric malady, afflicts a substantial global demographic, engendering considerable disease burden due to its elevated morbidity and mortality rates. Contemporary therapeutic approaches for depression encompass the administration of serotonin reuptake inhibitors, monoamine oxidase inhibitors, and tricyclic antidepressants, albeit these pharmaceuticals potentially induce adverse neurological and gastrointestinal effects. Traditional Chinese Medicine (TCM) natural products proffer the benefits of multi-target, multi-level, and multi-channel depression treatment modalities. In this investigation, we conducted a comprehensive literature review of the past 5 years in PubMed and other databases utilizing the search terms “Depression,” “Natural medicines,” “Traditional Chinese Medicine,” and “hypothalamic–pituitary–adrenal axis.” We delineated the 5 most recent and pertinent signaling pathways associated with depression and hypothalamic–pituitary–adrenal (HPA) axis dysregulation: nuclear factor kappa light-chain-enhancer of activated B cell, brain-derived neurotrophic factor, mitogen-activated protein kinase, cyclic AMP/protein kinase A, and phosphoinositide 3-kinase/protein kinase B. Additionally, we deliberated the antidepressant mechanisms of natural medicines comprising alkaloids, flavonoids, polyphenols, saponins, and quinones via diverse pathways. This research endeavor endeavored to encapsulate and synthesize the progression of TCMs in modulating HPA axis-associated signaling pathways to mitigate depression, thereby furnishing robust evidence for ensuing research in this domain.

## 1. Introduction

Depression is a common psychological disorder characterized by symptoms, such as low mood, loss of interest and pleasure, sleep disturbances, changes in appetite, problems with attention, and self-evaluation, which significantly affect the quality of life.^[1]^ Depression is the third leading cause of disease burden worldwide and is expected to rank first by 2030. More than 350 million people worldwide are affected by depression, and the number of disability-adjusted life years caused by depressive disorders represents 1.84% of the world population. Depression is characterized by high morbidity, disability rate, and mortality.^[2,3]^ Depression is not merely a functional psychiatric disorder but a complex illness involving genetic, psychological, biochemical, and social-environmental factors.^[4,5]^ Consequently, various hypotheses for the pathogenesis of depression have been proposed from different perspectives.

As early as the 1950s, many scholars discovered that depression onset was associated with a decrease in the levels of norepinephrine (NE) and dopamine.^[6]^ Subsequently, selective 5-hydroxytryptamine (5-HT) reuptake inhibitors were discovered to improve depressive symptoms by inhibiting the reuptake of 5-HT and increasing its concentration in the synaptic cleft. Since then, the monoamine neurotransmitter theory of depression has been widely accepted; the theory hypothesizes that the etiology of depression originates from a defect in monoaminergic neurotransmission. By the beginning of the 21st century, the HPA axis function was found to be often abnormal in patients with depression.^[7–10]^ Under normal circumstances, activation of the HPA axis leads to the hypothalamus releasing corticotropin-releasing hormone (CRH), which in turn stimulates the pituitary to release adrenocorticotropic hormone (ACTH), ultimately leading to the synthesis and release of glucocorticoids such as cortisol (CS) and corticosterone (CORT). The release of CS inhibits the secretion of CRH and ACTH from the hypothalamus and pituitary gland, respectively, forming a negative feedback regulatory loop. However, overactivation of the HPA axis in patients with depression can lead to excessive release of CRH and CS. Long-term high levels of CRH and CS can damage the structure and function of the temporal lobe and hippocampus, reduce the synaptic plasticity of the hippocampal dentate gyrus, cause atrophy and regenerative dysfunction of pyramidal neurons, and result in temporal lobe atrophy and hippocampal deficits, ultimately manifesting as depressive symptoms. In addition, abnormal HPA axis function may lead to a neurotransmitter imbalance. Hyperactivity of the HPA axis can induce the expression of tryptophan decarboxylase and transaminase, which degrade the 5-HT precursor tryptophan and NE precursor tyrosine in the blood, thereby reducing the synthesis of 5-HT and NE neurotransmitters and inducing depression-like behavior.^[11]^ These data suggest that the HPA axis hypothesis of depression interacts with the neurotransmitter hypothesis.

**Figure 1.**
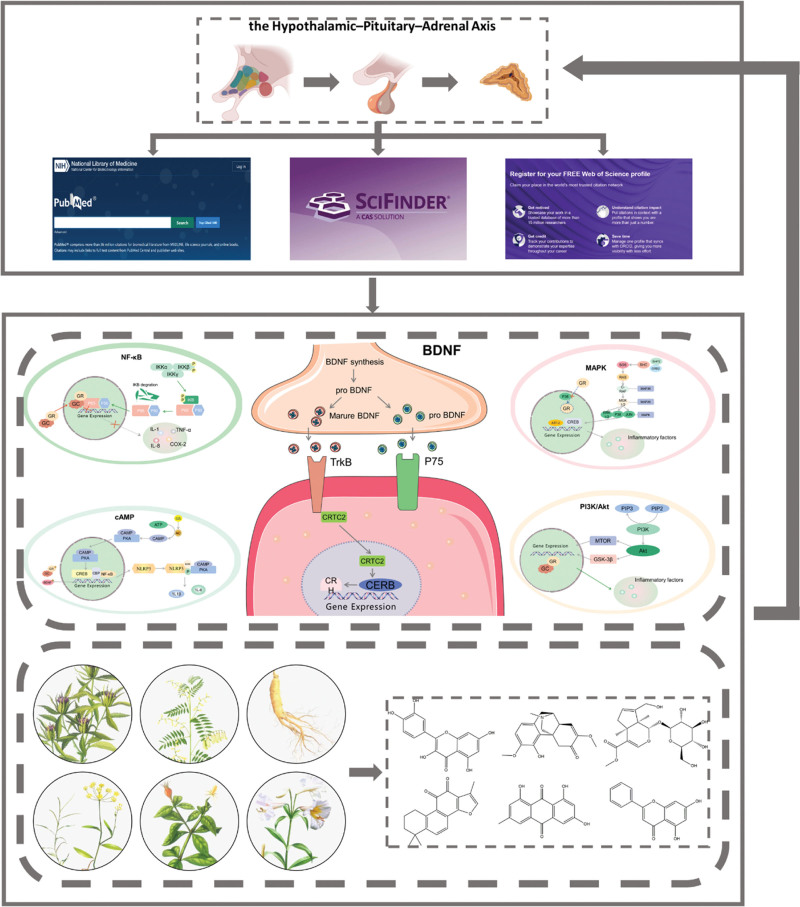
Flow chart.

The goal of treating depression is to alleviate symptoms, improve patients’ quality of life, and prevent relapse. Limited types of antidepressant drugs are currently available. Common antidepressant medications include Selective Serotonin Reuptake Inhibitors, tricyclic antidepressants, and Monoamine Oxidase Inhibitors, among others. These medication effects may take several weeks or even months to manifest, potentially leading to patient dissatisfaction and tolerance issues. Antidepressant medications may cause side effects, such as sexual dysfunction, weight gain, and cardiovascular problems.^[12,13]^ Traditional Chinese medicine (TCM) has a history of thousands of years, and its methods for treating depression have been applied in clinical practice.^[14]^ For example, the classic TCM formula Xiaoyaosan is commonly used to treat depression. Multiple meta-analyses have shown that the oral administration of Xiaoyaosan can benefit patients with depression and alleviate accompanying symptoms.^[15,16]^ Moreover, various TCM compound formulas, such as Guipi Tang,^[17]^ Ganmai Dazao Tang,^[18]^ and Shugan Jieyu Capsules,^[19]^ have shown good efficacy in the clinical treatment of depression. However, compared to that of conventional drug treatments, the efficacy and safety of herbal medicines lack sufficient scientific evidence, limiting their application in clinical practice. Therefore, we conducted a literature review by searching the PubMed, SciFinder, and Web of Science databases to summarize the HPA axis-related signaling pathways involved in the pathogenesis of depression and reviewed the latest progress in using natural products to target these pathways for the treatment of depression (Fig. 1).

## 2. Classical signaling pathway

**Figure 2.**
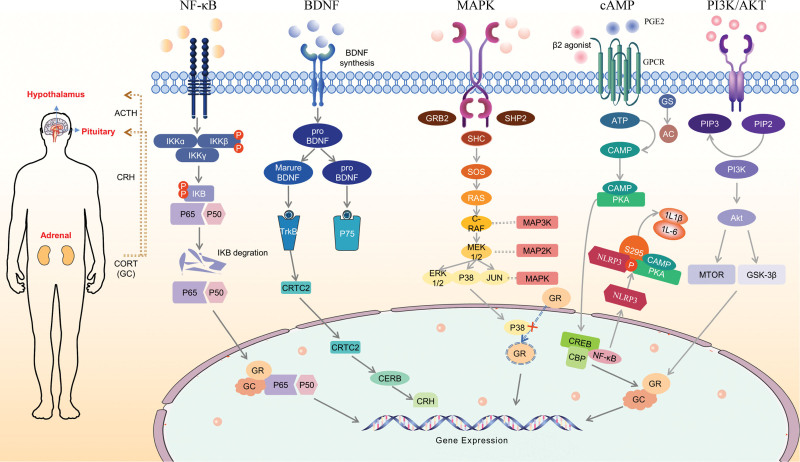
Signal pathways related to the HPA axis that affect depression. HPA = hypothalamic-pituitary-adrenal.

The onset and development of depression are influenced by processes such as the HPA axis, inflammatory response, monoaminergic system, brain-derived neurotrophic factor levels, and oxidative stress.^[20]^ Abnormal activation of different signaling pathways can directly or indirectly interfere with different stages of depression (Fig. 2).

### 2.1. Nuclear factor kappa light-chain-enhancer of activated B cell (NF-κb) signaling pathway

NF-κB is a transcription factor comprised of homodimeric or heterodimeric subunits from the Rel family, including p50, p52, p65 (Rel-A), c-Rel, and Rel-B. The activation of the NF-κB pathway is initiated by the activation of the IκB kinase complex.^[21]^ Typically, NF-κB forms a stable cytoplasmic trimer by binding to dimeric IκB. When signals from various external stimuli are transmitted to the cytoplasm through cell membrane receptors, they can activate NF-κB by triggering the IκB kinase complex. This complex undergoes phosphorylation, ubiquitination, and hydrolysis by proteases.^[22]^

The NF-κB pathway plays a crucial role in regulating the HPA axis in the context of depression. Elevated levels of glucocorticoids (GC) in the HPA axis activate the promoter of the IκB-α gene, resulting in increased transcription of the IκB-α gene. Since the protein encoded by the IκB-α gene acts as an inhibitor of NF-κB, NF-κB remains sequestered in the cytoplasm and cannot translocate into the nucleus to exert its transcriptional function.^[23]^ Interestingly, NF-κB and glucocorticoid receptors (GR) may interact as functional transcriptional antagonists. Activation of NF-κB is inhibited, leading to increased transcription of GR, thereby reducing elevated GC levels and modulating the overexpression of the HPA axis.^[24]^ It is worth noting that the IκB kinase plays a pivotal role in the cellular signaling network. Its inhibition of hippocampal neuronal apoptosis through the activation of NF-κB may represent an effective mechanism for antidepressant action.^[25]^

### 2.2. Brain-derived neurotrophic factor (BDNF) signaling pathway

BDNF belongs to the neurotrophic factor family, which also includes nerve growth factor (NGF), neurotrophic factor-3 (NT-3), and NT-4/5. Initially synthesized in the endoplasmic reticulum as a precursor protein (proBDNF), BDNF undergoes cleavage by calcium-dependent serine proteases in the Golgi and endoplasmic reticulum to form its mature, active form, mBDNF. mBDNF binds to its high-affinity cell membrane receptor, tropomyosin receptor kinase B (TrkB), triggering downstream signaling pathways that facilitate neuronal growth, differentiation, synaptic stabilization, and long-term potentiation. In contrast, mBDNF inhibits neuronal excitation, reduces synaptic transmission efficiency, and promotes long-term synaptic potentiation through its interaction with its low-affinity receptor, p75NTR.^[26]^

In depression, there exists bidirectional regulation between the hyperactivity of the HPA axis and BDNF expression. Upon activation of the HPA axis, GR binds to GC and translocates to the nucleus, where they target the BDNF promoter, thereby driving the production of basal BDNF. When the HPA axis is overactive and GC levels increase, this leads to reduced BDNF expression and impairs the function of regions like the hippocampus. Consequently, these regions are unable to promote synaptic growth or maintain neuronal survival, resulting in a disruption of corresponding brain functions and the onset of depression.^[27]^ Changes in BDNF levels can also feed back into the HPA axis. Additionally, BDNF can regulate GR function by influencing GR nuclear translocation through a crosstalk mechanism.^[28]^ Elevated GR levels stimulate the excitation of corticotropin-releasing hormone (CRH) neurons, subsequently leading to increased CRH secretion. Moreover, neuropeptide W (NPW) plays a significant role in neuroendocrine regulation, and BDNF induces NPW mRNA expression, thereby enhancing CRH release.^[29]^ It is noteworthy that the differential regulation of CRH is dependent on the cAMP response element-binding protein (CREB)-regulated transcriptional coactivator-2 (CRTC2). CRTC2 maintains the equilibrium between BDNF and GC and regulates the HPA axis in the context of depression.^[30]^

### 2.3. Mitogen-activated protein kinase (MAPK) signaling pathway

The MAPK signaling pathway plays a vital role in regulating cell growth, differentiation, stress responses, and inflammation by responding to extracellular stimuli. The MAPK family primarily comprises extracellular signal-regulated kinases (ERK) 1, 2, and 5; Jun N-terminal kinase (JNK) 1, 2, and 3; and the p38 (α, β, γ, and δ) families.^[31]^ ERK is initiated by receptor-ligand interactions and sets off a cascade of reactions. Initially, extracellular factors bind to cell-surface receptors, promoting the aggregation and activation of Raf. Activated Raf, in turn, phosphorylates and activates MAPK kinase (MEK) 1/2, leading to the activation of ERK1/2. ERK1/2 exerts its biological effects primarily through gene transcription.^[32]^ Importantly, GR serves as a downstream substrate of ERK, and the activation of ERK can influence its translocation from the cytoplasm to the nucleus by phosphorylating a specific site on GR. This process may result in resistance to GC.^[33]^ Therefore, the hyperfunction of the HPA axis continues or is further aggravated, which is the most direct physiological and biochemical basis for inducing depression.

JNK is involved in cell differentiation, apoptosis, and stress response processes in the body. Under stressful conditions, the hypothalamus is initially activated, which promotes the transcription of c-Fos and c-Jun in the paraventricular nucleus of the hypothalamus. Subsequently, there is an expression of Fos and Jun proteins, leading to the formation of activator protein-1 (AP-1).^[34]^ AP-1 then stimulates the transcription of corticotropin-releasing factor (CRF) mRNA, thereby initiating the activation of the HPA axis.^[35]^ When stress persists, JNK becomes responsible for phosphorylating c-Jun, which in turn initiates apoptosis. This process is closely associated with apoptosis observed in hippocampal neurons in depression. Consequently, it results in elevated levels of ACTH, corticotropin (CORT), CRH, and HPA axis overactivity, ultimately triggering depression.^[36]^

Phosphorylation of p38MAPK under stress augments the transcriptional activity of the AP-1 family, influencing the expression of downstream CRH genes and modulating the activity of the HPA axis.^[37,38]^ Additionally, p38MAPK boosts CREB transcription, eliciting inflammatory responses that can harm central neurons, attenuate central reward feedback mechanisms, and dampen positive emotions.^[39]^ Concurrently, proinflammatory factors activate p38MAPK, which hinders GR nuclear translocation and results in abnormal GR phosphorylation, thereby exacerbating the progression of depression.^[40]^

### 2.4. Cyclic AMP (cAMP) signaling pathway

cAMP, a pivotal secondary messenger molecule in cells, exerts regulation over a multitude of cellular functions. These functions include neuronal growth and development, synaptic plasticity, neurogenesis, and the modulation of gene and protein expression. Additionally, cAMP possesses immunosuppressive and anti-inflammatory properties. The cAMP-dependent pathway is initiated by G protein-coupled receptors upon activation by extracellular ligands, such as β2 adrenergic receptor agonists and Prostaglandin Estradiol (E2).^[41]^ Within this context, CRH activates adenylate cyclase, which catalyzes the conversion of ATP to cAMP.^[42]^

The cAMP signaling pathway serves to repair neuronal damage in the hippocampus during states of depression and fosters the survival, regeneration, and differentiation of hippocampal neurons.^[43]^ Moreover, the cAMP signaling pathway enhances CRH synthesis by phosphorylating CREB.^[44]^ Concurrently, CRH upregulates BDNF, facilitates GR transcription in hippocampal neurons, and mitigates neuronal damage by inducing substantial GC production.^[45,46]^ In contrast, CRH elevates neuronal excitability and elicits corresponding changes in hypothalamic neuronal phenotypes by triggering intracellular Ca2 + influx and increasing cAMP levels.^[47,48]^ In summary, cAMP plays a pivotal role in maintaining HPA axis activity and contributes to the treatment of depression.

### 2.5. Phosphoinositide 3-kinase (pi3k)/protein kinase B (AKT) signaling pathway

The PI3K/AKT signaling pathway plays a protective role in hippocampal and cortical neurons, enhancing hippocampal synaptic plasticity, mitigating depression, and reducing psychiatric disorders. PI3K, a critical molecule within the growth factor superfamily signaling pathway, catalyzes the conversion of phosphatidylinositol 4,5-bisphosphate to phosphatidylinositol 3,4,5-trisphosphate. Phosphatidylinositol 3,4,5-trisphosphate binds to the pleckstrin homologous domain of downstream target Akt, facilitating the translocation of Akt from the cytoplasm to the cell membrane. This transition induces conformational changes in Akt, ultimately leading to its activation.^[49]^ There exist 3 isoforms of Akt (Akt1, Akt2, and Akt3); notably, Akt1 is associated with the severity of depression in individuals with depressive disorders.^[50]^

PI3K functions upstream of GR transcriptional activity. Upon binding to GC in the cytoplasm, GR relocates to the nucleus, recognizes hormone response elements within DNA, reduces serum CS levels, and diminishes the excitability of the HPA axis.^[51]^ Additionally, the activation of PI3K/Akt by BDNF enhances the mRNA expression of neuropeptide W (NPW) in hypothalamic neurons. NPW serves as a stress mediator in the hypothalamus, stimulating corticotropin-releasing hormone (CRH) expression and activating the HPA axis.^[29]^ Of note, microglia activate the PI3K/AKT signaling pathway to shift from their M1 phenotype, leading to HPA axis hyperactivity, neurotransmitter dysfunction, and the production of proinflammatory mediators and oxidants. Subsequently, microglia polarize towards the M2 phenotype, exerting antidepressant effects.^[52]^

## 3. Natural medicines for depression

TCM has been used to treat depression with significant efficacy. Consequently, this study seeks to comprehensively examine the mechanisms of action attributed to various active constituents found in TCM that contribute to the treatment of depression by modulating HPA axis-related pathways. This review aims to serve as a valuable reference for future in-depth research and clinical assessments. The chemical structures of natural medicines are depicted in Table 1.

**Table 1:**
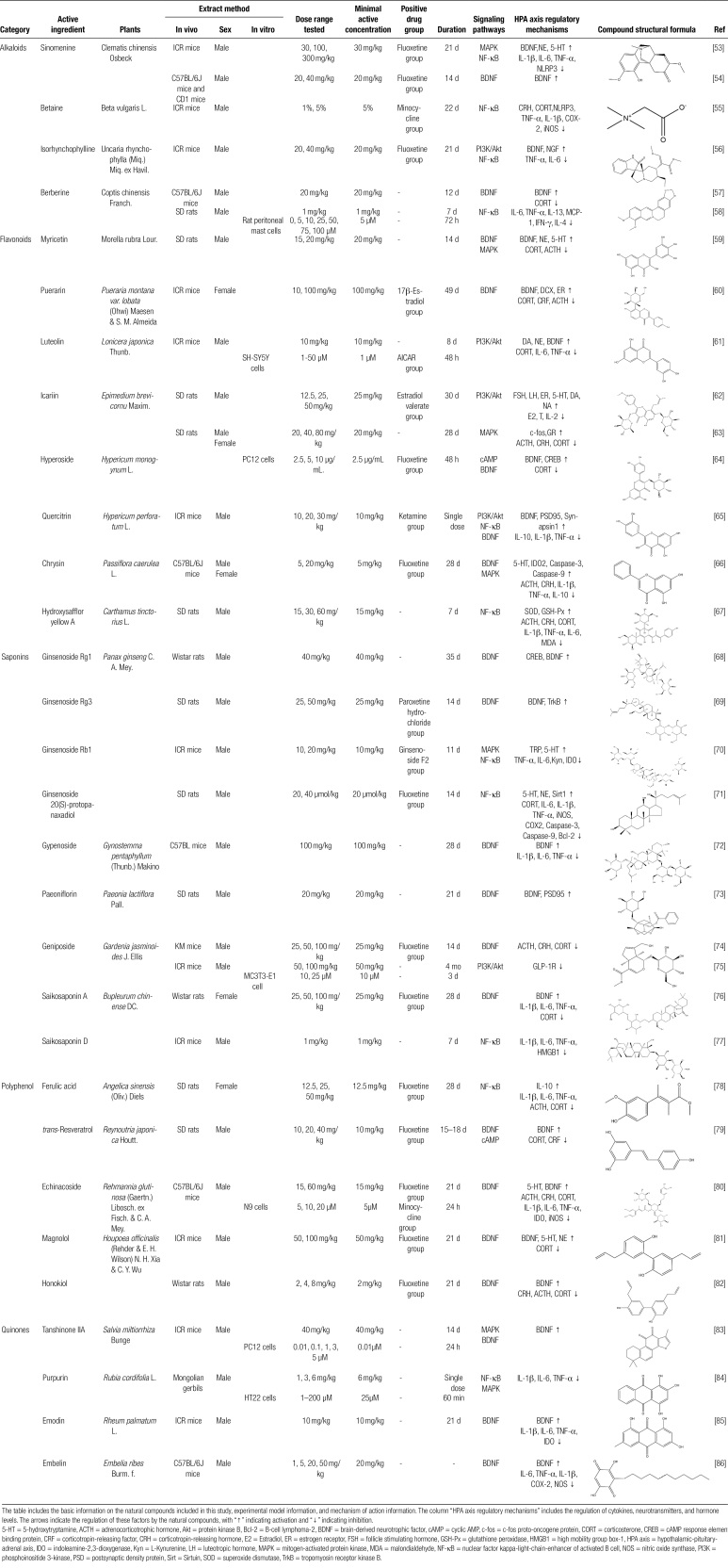
Regulatory effects of TCM natural compounds on depression-related signaling pathways.

### 3.1. Alkaloids

Alkaloids represent a widespread category of nitrogen-containing organic compounds, further classified into structural subtypes including piperidines, isoquinolines, quinolones, indoles, terpenes, and steroids. Alkaloids possess a versatile range of pharmacological properties, including antiviral, anti-cancer, immunosuppressive, anti-inflammatory, and analgesic effects.^[87,88]^ Emerging research has unveiled the antidepressant potential of alkaloids in response to psychological stress.^[89]^ Within alkaloids, the nitrogen heterocycle serves as the pivotal active group responsible for their efficacy in depression treatment. Notably, serotonin, a derivative of alkaloids, shares structural similarities with indole alkaloids, as both feature a fusion of a 6-membered heterocycle and a 5-membered ring. This structural resemblance provides robust evidence supporting the antidepressant properties of alkaloids.^[90]^ Sinomenine is a morphine-type, isoquinoline-derived alkaloid isolated from *Clematis chinensis* Osbeck. Studies have shown that sinomenine restores norepinephrine and 5-HT synthesis in the hippocampus of depressed mice by inhibiting the MAPK/NF-κB pathway.^[53]^ Interestingly, betaine can also exert antidepressant effects by inhibiting the NF-κB pathway. In contrast to the antidepressant mechanism of sinomenine, inhibition of the NF-κB pathway by betaine can promote the polarization of M1 microglia into M2, leading to a decrease in CRH and serum CORT concentration levels in mice, thus reversing HPA axis hyperactivity.^[55]^ Moreover, sinomenine promotes the synthesis of 5-HT by activating the BDNF signaling cascade in the brain.^[54]^ Isorhynchophylline is a hydroxyindole alkaloid isolated from *Uncaria rhynchophylla* (Miq.) Miq. ex Havil not only modulates the PI3K/Akt pathway but also increases glycogen synthase kinase-3β (Ser^9^) and Akt (Ser^473^) phosphorylation levels. It also upregulates BDNF levels in the hippocampus and cerebral cortex of mice and decreases the transport of NF-κB p65 and the binding activity of NF-κB in the nucleus.^[56]^ These mechanisms play an important role in improving depression-like behavior by isorhynchophylline. Berberine restores negative feedback hyperactivity of the HPA axis by activating BDNF/CREB signaling to upregulate hippocampal monoamine levels and downregulate serum CORT levels.^[57]^ Simultaneously, berberine exerts antidepressant effects by inhibiting the release of the NF-κB signaling pathway and proinflammatory cytokines.^[58]^

### 3.2. Flavonoids

Numerous plants biosynthesize flavonoids as secondary metabolites. Flavonoids encompass aromatic carbon rings and benzo-γ-pyranone parent nuclei, with chemical subcategories including isoflavones, flavanones, flavanols, and cyanidin chlorides.^[91]^ Flavonoids exhibit a wide spectrum of pharmacological effects, encompassing anti-depressive, anti-inflammatory, anti-cancer, anti-viral, anti-epileptic, and antioxidant properties.^[92,93]^ The manifestation of these effects is contingent upon the specific type and dosage of flavonoids employed.^[94]^ Notably, flavonoids offer robust physiological activity, minimal toxicity, and reduced side effects. Alterations in the hydroxyl group within the flavonoid chemical structure have been observed to elevate BDNF levels in the brain, heighten 5-HT and NE concentrations, scavenge free radicals, and confer antioxidant capabilities. Consequently, flavonoids are frequently utilized as natural remedies for depression.^[95,96]^ Furthermore, a substantial body of evidence supports the capacity of various flavonoids to diminish CORT levels in the hippocampus. For example, myricetin inhibits the secretion of CORT and ACTH by phosphorylating ERK and increasing the expression of BDNF and TrkB. It restores the decreased serotonin levels in the fear circuit area, medial prefrontal cortex, and hippocampus of rats.^[59]^ Puerarin, a natural isoflavone derived from *Pueraria montana var. lobata* (Ohwi) Maesen & S. M. Almeida, attenuates serum CORT levels and affects neuronal cell migration by upregulating the expression of CS and BDNF.^[60]^ Both luteolin and icariin regulate hormone levels and immune function by activating the PI3K/Akt signaling pathway.^[61,62]^ Furthermore, icariin plays a role in correcting adverse morphological changes in neuronal dendrites in the hippocampus. This mechanism is mediated by its ability to inhibit ACTH, CRH, and CORT by increasing GR expression, which leads to ERK and CREB phosphorylation.^[63]^ Hyperoside reverses the effect of CORT by activating the cAMP and BDNF signaling pathways and upregulating CREB levels.^[64]^ These flavonoids can effectively correct HPA axis dysregulation and monoamine neurological imbalance after reducing CORT levels in serum and exerting antidepressant effects. Quercitrin and chrysin improve CREB phosphorylation and BDNF signaling in the hippocampus and prefrontal cortex of rats and improve neuroplasticity.^[65,66]^ Hydroxysafflor yellow A exerts inhibitory effects on HPA signaling, hippocampal inflammation, and oxidative stress in depressed rats by inhibiting NF-κB signaling pathway effects.^[67]^

### 3.3. Saponins

Saponins constitute a class of high-molecular-weight glycosides derived from triterpenoids or steroids. They are further categorized into steroidal and triterpenoid saponins based on structural distinctions. Research has substantiated their multifaceted pharmacological properties, including anti-inflammatory, antioxidant, anti-apoptotic, anti-diabetic, anti-cancer, and neuroprotective effects.^[97–99]^ It worth noting that their biological activity is influenced by factors such as glycosidic structure, monosaccharide composition, and sugar chain structure. The latest clinical reports have shown the potential role of saponins in the clinical treatment of depression.^[85]^ Ginseng is rich in several saponins, some of which are involved in the regulation of the HPA axis and neuroplasticity and exert neuroprotective and antidepressant effects. For example, ginsenosides Rg1 and Rg3 can upregulate the expression of BDNF and regulate monoamine imbalance in the medial prefrontal cortex and hippocampus.^[68,69]^ Ginsenoside Rb1 inhibits NF-κB transcriptional activity by downregulating the MAPK signaling pathway, reduces the release of proinflammatory cytokines, and promotes normal GR expression in the nucleus, further reducing HPA axis hyperactivity.^[70]^ Ginsenoside 20(S)-protopanaxadiol can also reduce the levels of proinflammatory cytokines in serum by inhibiting the NF-κB signaling pathway. Furthermore, ginsenoside 20(S)-protopanaxadiol mediates neuronal apoptosis by inhibiting microglial activation, thus improving HPA axis dysfunction.^[71]^ In addition to ginsenosides, other saponins in TCM have antidepressant effects. Gypenoside, paeoniflorin, and geniposide can correct HPA disorders by upregulating BDNF expression and activating the downstream cascade TrkB/ERK/CREB.^[72–74]^ Geniposide also inhibits hippocampal neuronal apoptosis by activating the PI3K/Akt signaling pathway.^[75]^ Both saikosaponin A and saikosaponin D in *Bupleurum chinense* DC can regulate the negative feedback of the HPA axis and inhibit the release of proinflammatory factors.^[76,77]^

### 3.4. Polyphenols

Polyphenolic compounds are naturally-occurring secondary metabolites found in plants. They feature one or more phenolic hydroxyl groups within their chemical structure, rendering them biologically active small-molecule phenolic compounds. These polyphenol compounds possess the capacity to directly participate in the scavenging of free radicals by acting as hydrogen donors, making them natural antioxidants. A growing body of research has revealed the neuroprotective attributes of polyphenolic compounds and their role in bolstering cognitive function.^[100–102]^ Notably, the presence of carboxyl and carbonyl groups in their structure plays a pivotal role in determining their antidepressant activity. Ferulic acid modulates the abnormal HPA axis by increasing GR protein expression and decreasing ACTH and CORT concentrations. Meanwhile, ferulic acid downregulates NF-κB activation and inhibits inducible nitric oxide synthase overexpression in hippocampal neurons, repairing neuronal damage caused by inflammation and oxidative stress and improving depression-like behavior.^[78]^ *Trans*-resveratrol, a natural polyphenol found in grape seeds and grape skins, also upregulates GR expression in the hypothalamus and hippocampus, contributing to a reduced adrenal index and CRF levels^[79]^ and can inhibit the secretion of inflammatory factors in N9 cells and the polarization of microglia M1, improving damaged hippocampal nerves.^[80]^ Magnolol and its isomer honokiol are polyphenolic compounds isolated from magnolia, both of which have been shown to increase neurotransmitter levels in the hippocampus and normalize the function of the HPA axis, mainly by activating CREB phosphorylation and upregulating BDNF levels.^[81,82]^ Interestingly, *trans*-resveratrol and echinacoside also improve the HPA axis through this mechanism in the treatment of depression.

### 3.5. Quinones

Quinones are natural organic compounds characterized by an unsaturated cyclic dione structure within the molecule or the ability to readily convert to such a structure. They are classified into 4 primary categories: benzoquinones, naphthoquinones, phenanthrenequinones, and anthraquinones.^[103]^ Quinone compounds display diverse biological activities, encompassing antibacterial, anti-inflammatory, antiviral, tumor growth inhibition, and antidepressant effects.^[104,105]^ In many instances, quinones serve as crucial electron transport mediators within biological organisms, facilitating reversible redox processes. Alterations in the functional groups of quinone structures can yield active and selective ligands for various biological targets, allowing them to bind or interact with a range of biological receptors. Consequently, quinones offer a broad spectrum of biological activities and opportunities for structural modifications.^[106]^ The antidepressant effects of tanshinone IIA are mediated by the ERK/CREB/BDNF pathway. It not only increases the dendritic complexity and dendritic spine density of neurons in the hippocampus and prefrontal cortex but also upregulates BDNF and phosphorylated TrkB levels in the hippocampus.^[83]^ Purpurin significantly decreases the expression of NF-κB signaling and MAPK phosphorylation in astrocytes and protects damaged neurons.^[84]^ In addition, purpurin inhibits HPA axis overactivity by decreasing serum ACTH and CORT levels, resulting in antidepressant effects.^[107]^ Emodin is an anthraquinone compound found in *Rheum palmatum* L that can alleviate GR hypoexpression and increase BDNF expression levels.^[85]^ Embelin modulates negative feedback on the HPA axis by increasing BDNF expression. Importantly, embelin not only increases the expression of catalase to prevent oxidative stress in the brain but also decreases the expression of proinflammatory cells to prevent neuronal inflammation.^[86]^

## 4. Clinical application

The effectiveness of TCM and its natural products in treating depression has gained increasing recognition. Many researchers have confirmed this result using TCM or effective ingredient extracts in clinical trials. A single-center, randomized, double-blind clinical trial included 60 patients with type 2 diabetes and depression. The experimental group received 700 mg *M. officinalis* extract capsules daily, whereas the control group received a placebo for 12 weeks. The results showed that taking *M. officinalis* extract significantly reduced the CORT levels and increased the γ-aminobutyric acid levels, effectively improving depression and anxiety.^[108]^ Another study used a 3-arm parallel randomized controlled trial to study the effects of Lavender and Chamomile essential oil aromatherapy on depression, anxiety, and stress in the elderly. Compared to the control group, patients showed significant improvements in depression, anxiety, and stress levels immediately and at 1 month after aromatherapy.^[109]^ A double-blind, placebo-controlled, randomized clinical trial included 52 women with mild-to-moderate depression. The experimental group received 15 mg saffron capsules twice daily, while the control group received the same dose of placebo capsules. Compared to the placebo, the average Beck Depression Inventory II depression score in the saffron group was significantly reduced.^[110]^ Another randomized clinical trial compared the clinical efficacy of saffron and sertraline, once again proved that saffron improved depression more significantly.^[111]^ Moreover, 45 patients with mild or moderate depression took tablets of Rhodiola and saffron extracts for 6 weeks, and the Hamilton Depression Scale (HAMD) score dropped significantly by 58%±28.5%, with good safety during treatment and no adverse reactions.^[112]^

In addition to improving various diseases with TCM alone, combined TCM treatments can increase efficacy and reduce adverse reactions compared to those associated with conventional drug treatments.^[113]^ A 6-week, randomized, double-blind, placebo-controlled trial involving 108 adult male individuals showed that supplementing 1000 mg of curcumin daily with escitalopram significantly reduced the HAMD and Montgomery-Asberg Depression Rating Scale scores. Further, patients taking curcumin had increased plasma BDNF levels, decreased IL-1β and TNF-α levels, and decreased salivary CS concentration.^[114]^ Another trial showed that adding curcuminoids to piperine combined with medication in conventional antidepressant treatment significantly reduced the HAMD and Beck Depression Inventory II total scores compared with those associated with conventional antidepressant treatment.^[115]^ This prospective, randomized, blinded endpoint trial evaluated the efficacy of combined chamomile and saffron tea treatments in patients with depression. The experimental group received herbal tea sachets twice daily (20 mg chamomile and 1 mg saffron/packet) based on the conventional medication for 1 month. Compared to the group receiving only conventional medication, the experimental group exhibited significantly upregulated plasma BDNF levels and reduced inflammatory marker CRP and TRP levels.^[116]^

## 5. Discussion

Recent research has underscored the significance of HPA axis dysregulation in the pathogenesis of depression. The intricate interplay of the NF-κB, BDNF, MAPK, cAMP, and PI3K/Akt signaling pathways forms a complex regulatory network during the development of depression. This study aimed to identify natural products with therapeutic potential for depression by modulating these pathways. Natural products, including alkaloids, flavonoids, polyphenols, saponins, and quinones, were screened for their antidepressant properties. The physiological activities of these natural products are intricately linked to their chemical structures, with the arrangement of parent atoms and substituents influencing their pharmacological effects. Thus, an in-depth analysis of the structure-activity relationships of these natural products can significantly enhance our ability to predict and optimize their efficacy, offering a foundational framework for the development of more potent depression treatments. Moreover, certain natural products, such as berberine, baicalin, and quercetin, demonstrate the potential to ameliorate depression-related comorbidities like insomnia, cognitive impairment, and sexual dysfunction. These effects are achieved through their actions on multiple signaling pathways and targets, as indicated by previous studies.^[117–119]^

Modern pharmacological studies have indicated that prolonged administration of natural products yields superior results in alleviating depressive-like behaviors in rats compared to single-dose administration.^[76]^ It worth noting that many natural products exhibit potent antidepressant effects at higher doses.^[55,72]^ However, determining the optimal drug concentration is crucial. For instance, in HT22 cells, treatment with various purine concentrations ranging from 1 to 200 μM revealed that 25 μM of purine exhibited the least toxicity.^[84]^ In 3T3-L1 adipocytes, concentrations of 50 μM and 100 μM purine demonstrated positive effects.^[120]^ Furthermore, the study on pinecone chrysanthemum glycosides in rats found that both 15 mg/kg and 60 mg/kg doses reversed HPA axis overexpression. However, the lower 15 mg/kg dose significantly increased the levels of 5-HT, NE, and DA, which was not observed with the higher 60 mg/kg dose.^[80]^ These findings emphasize that the optimal concentrations of natural products for achieving antidepressant effects can vary depending on the subject. Therefore, identifying the most suitable drug concentration is pivotal for guiding clinical medication. While most natural products are generally safe, it important to recognize that different biological species and administration routes can lead to the conversion of certain drugs into toxic substances during metabolism. For example, oral administration of 120 mg/kg of embelin to female rats over 6 consecutive weeks resulted in short-term toxicity, including liver and kidney collapse and necrosis, along with perinuclear vacuolation. These effects were alleviated upon discontinuation of embelin.^[121]^ Consequently, analyzing the toxicity of TCMs prior to clinical trials is essential. Strictly controlling the relatively narrow safety margin, while preserving efficacy, helps prevent cases of poisoning.^[122]^

Animal models serve as valuable tools for studying the pathogenesis and symptoms of human depression, allowing researchers to explore specific components of brain circuits that underlie psychopathology. However, it important to acknowledge the phenotypic differences that exist in clinical depression, and researchers must carefully consider the relationship between different models and phenotypes when selecting an appropriate depression model. Additionally, differences in sensitivity to particular stressors have been observed between animals of different sexes. For example, Wistar-Kyoto rats tend to exhibit more pronounced depressive-like behavior compared to SD rats, and within the Wistar-Kyoto rat model, females often display more pronounced depressive-like behavior than males.^[123]^ Conversely, chronic stress appears to have a lesser impact on female CD1 mice compared to males.^[124]^ These sex-specific differences exhibit a high degree of species specificity and are influenced by various social and cultural factors in humans. Notably, only 1 study in the review distinguished between male and female rats,^[63]^ with the majority explicitly selecting only male rats. To bridge the gap between animal models and clinical practice, future studies should consider including both male and female subjects in their research scope. Furthermore, while animal models provide valuable insights, there remains a disparity between findings in animal models and their application in clinical practice. Few clinical studies have explored the use of natural products targeting the HPA axis for depression treatment. There is a pressing need for more large-sample randomized or placebo-controlled clinical trials in this regard.

At present, many natural products used for the treatment of depression are sourced from plants and contain active ingredients. However, these plant-derived compounds often face significant challenges, including low bioavailability, limited pharmacological activity, and rapid metabolic degradation. These issues can impede the translation of plant-derived natural products from basic research to clinical applications. To overcome these hurdles, there is a potential avenue of designing and synthesizing chemical analogs that can inhibit rapid chemical degradation. Additionally, formulating these compounds into nanoparticles, liposomes, or phospholipid complexes can enhance their targeting and effectiveness.^[125]^ It worth noting that the chemical composition of plant secondary metabolites can be influenced by various factors such as nutrient availability, climate conditions, and ecological factors. Therefore, there is a need for rigorous quality control measures to be implemented for natural TCMs. Establishing a hierarchical, graded standardization scheme is essential to ensure the consistency and reliability of these products.^[126]^ While this study primarily focuses on the compounds found in TCM, it is imperative to further explore the mechanisms underlying the therapeutic effects of TCM compounds in treating depression by leveraging modern technology. Clear diagnostic criteria and efficacy standards should be established to meet the growing demand for TCM in the market and to provide a solid scientific foundation for innovative approaches to depression treatment. Moreover, enhancing collaboration between traditional medicine practitioners and healthcare professionals is of paramount importance. This collaboration can ensure the coordinated and comprehensive care of patients dealing with depression, combining the strengths of traditional medicine with evidence-based practices for improved patient outcomes.

## Author contributions

**Writing – original draft:** Jiawen Liu, Tianwei Meng, Guangyu Cheng.

**Writing – review & editing:** Chaojie Wang, Weiping Cheng, Qi Zhang.
